# Increased Albuminuria Is Independently Associated with Peripheral Neuropathic Pain in Patients with Type 2 Diabetes

**DOI:** 10.3390/medsci14060605

**Published:** 2026-09-25

**Authors:** Chun-Yan Hsu, I-Te Lee

**Affiliations:** 1 Department of Education, Taipei Veterans General Hospital, Taipei 112201, Taiwan; sophia12281228@gmail.com; 2 Division of Endocrinology and Metabolism, Department of Internal Medicine, Taichung Veterans General Hospital, Taichung 407219, Taiwan; 3 School of Medicine, National Yang Ming Chiao Tung University, Taipei 112304, Taiwan; 4 School of Medicine, Chung Shan Medical University, Taichung 402306, Taiwan; 5 Department of Post-Baccalaureate Medicine, College of Medicine, National Chung Hsing University, Taichung 402002, Taiwan

**Keywords:** chronic kidney disease, neuropathy, pain, diabetes, urinary albumin-to-creatinine ratio, Identification Pain, Douleur Neuropathique en 4, questionnaire

## Abstract

**Background**: Peripheral neuropathic pain is prevalent in patients with type 2 diabetes (T2D). This retrospective cross-sectional study aimed to examine the association between urinary albumin-to-creatinine ratio (UACR) and peripheral neuropathic pain in patients with T2D. **Methods**: Medical information was retrospectively collected from patients who underwent assessments of Identification Pain (ID Pain) and Douleur Neuropathique en 4 (DN4) questionnaires. Reduced estimated glomerular filtration rate (eGFR) was defined as an eGFR value < 60 mL/min/1.73 m^2^. **Results**: The prevalence of ID Pain scores ≥ 2 was significantly higher in the reduced eGFR group than the preserved eGFR group (*p* < 0.001). The prevalence of ID Pain scores ≥ 2 showed a positive trend from normal UACR to moderately and severely increased albuminuria in both the preserved eGFR (15.9%, 27.0%, and 33.8%; *p* < 0.001) and reduced eGFR (25.6%, 26.1%, and 37.1%; *p* = 0.035) groups. In multivariable regression analyses, the risk of ID Pain scores ≥ 2 significantly increased in the group with severely increased albuminuria (adjusted odds ratio: 2.016, 95% confidence interval: 1.471–2.763; *p* < 0.001) compared to the normal UACR group; however, reduced eGFR became not significantly associated with peripheral neuropathic pain (*p* = 0.466) after adjusting for confounding factors. Similar findings were replicated for peripheral neuropathic pain defined as DN4 scores ≥ 4. **Conclusions**: Peripheral neuropathic pain was significantly associated with increased albuminuria in both the reduced eGFR and preserved eGFR groups. However, a reduced eGFR was not independently associated with peripheral neuropathic pain.

## 1. Introduction

Diabetes mellitus (DM) is a major cause of mortality and disability worldwide [1]. A report from the International Diabetes Federation (IDF) Diabetes Atlas 11th edition stated that 589 million people aged between 20 and 79 years were living with DM [2]. Moreover, the number of affected patients is estimated to reach 853 million globally by 2050, and this trajectory underscores a critical public health burden [3]. In parallel with the global trajectory, the proportion of patients with DM in Taiwan increased by 66% from 2005 to 2014 among adults aged between 20 and 79 years [4]. Type 2 diabetes (T2D) is the dominant type of DM contributing to this increasing trend in the number of affected patients [4].

A previous meta-analysis reported a prevalence of 31.5% for diabetic peripheral neuropathy (DPN) [5]; however, its prevalence can vary depending on the diagnostic criteria [6]. Based on a diabetes registry of the Canadian population, 26% of patients with T2D had DPN, as assessed using the modified Toronto Clinical Neuropathy score [7]. In a Japanese nationwide survey, 27.7% of patients with T2D were diagnosed with DPN, and 61.5% of those with DPN had sensory neuropathy based on clinical symptoms and signs [8]. Furthermore, neuropathic pain is prevalent in patients with T2D and sensory neuropathy [9]. Among outpatients with T2D in Myanmar, 580 (63.8%) of 909 patients with sensory neuropathy had neuropathic pain [10]. In a Korean nationwide cohort, 43.1% of patients with T2D and DPN had neuropathic pain [11]. Limited daily activities have been reported in 85% of patients with peripheral neuropathic pain [12]. Peripheral neuropathic pain has therefore become an important focus in patients with T2D due to its increased health and economic burden [13].

An increased urinary albumin-to-creatinine ratio (UACR) has been reported to be associated with asymptomatic DPN with an abnormal nerve conduction velocity (NCV) in Chinese patients with T2D [14]. However, nephropathy, defined as UACR > 30 mg/g, was not significantly associated with sensory neuropathy in Japanese patients with T2D and DPN [8]. Nephropathy was also not significantly associated with neuropathic pain in Korean patients with T2D and DPN [11]. As peripheral neuropathic pain involves complex mechanisms with uncertain abnormalities in the somatosensory nervous system, the associated factors and treatment for peripheral neuropathic pain are not the same as those for other types of DPN [15]. Therefore, we aimed to examine the association between the UACR and peripheral neuropathic pain in patients with T2D.

## 2. Materials and Methods

### 2.1. Study Design and Patients

This retrospective cross-sectional study was conducted at Taichung Veterans General Hospital, and all data were collected retrospectively. This study was approved by the Institutional Review Board of Taichung Veterans General Hospital (TCVGH-IRB No. CE21376A, approval date 27 October 2021). The need for informed consent was waived owing to the retrospective study design and the removal of identifying patient information.

Details regarding the study design have been reported previously [16]. Briefly, outpatients diagnosed with DM were encouraged to participate in the Diabetes Pay-for-Performance program, and an annual comprehensive assessment for chronic diabetic complications including DPN was requested [17]. We retrospectively collected the data of patients who had completed the Identification Pain (ID Pain) questionnaire and the Douleur Neuropathique en 4 (DN4) questionnaire between January 2013 and October 2013. We excluded patients based on the following criteria: (1) DM other than T2D, as well as current pregnancy; (2) active lesions of the DM foot; (3) renal replacement therapy or an estimated glomerular filtration rate (eGFR) < 5 mL/min/1.73 m^2^; (4) any condition unsuitable for the study including cancer, current use of medication for pain control, and drug or alcohol abuse; (5) incomplete annual comprehensive assessment. For patients who were repeat-enrolled during the study period, only the results from the last assessment of peripheral neuropathic pain with complete information were collected.

### 2.2. Peripheral Neuropathic Pain Assessments

ID Pain is a self-reported questionnaire comprising six items. The scoring algorithm assigns 1 point for each positive response to items 1–5 and deducts 1 point for a positive response to item 6. The total score for ID Pain ranges from −1 to 5, and a threshold of ≥ 2 was used to identify peripheral neuropathic pain. The DN4 questionnaire is a clinical assessment list performed by educators credentialed by the Taiwanese Association of Diabetes Educators. This 10-item instrument employs a binary scoring system (1 point for a positive response to each item), and a threshold cumulative score of ≥ 4 out of 10 was used to define peripheral neuropathic pain. Taiwanese versions of both the ID Pain and DN4 questionnaires have been validated [18,19]. Our previous report validated the reproducibility of the questionnaires [16]. Briefly, Pearson correlation revealed highly positive correlations between two repeated assessments using the same questionnaires, and Bland–Altman plots revealed results lying within limits of agreement. In the present study, the primary aim was to examine the independent association between UACR and peripheral neuropathic pain defined as ID Pain scores ≥ 2. We performed the same statistical analyses to confirm the association between UACR and DN4 scores ≥ 4.

### 2.3. Laboratory Assessments

Laboratory information was retrospectively collected from the annual comprehensive assessment of the Diabetes Pay-for-Performance program. The biochemical data of the blood samples included plasma glucose, glycated hemoglobin (HbA1c), serum creatinine, glutamate pyruvate transaminase (GPT), total cholesterol, low-density lipoprotein (LDL) cholesterol, high-density lipoprotein (HDL) cholesterol, and triglycerides. Urine albumin and creatinine levels were recorded to calculate the UACR. A normal UACR was defined as UACR < 30 mg/g, moderately increased albuminuria was defined as UACR between 30 and 299 mg/g, and severely increased albuminuria was defined as UACR ≥ 300 mg/g [20]. The eGFR was calculated using the Chronic Kidney Disease Epidemiology Collaboration 2021 creatinine race-free equation [21]. A reduced eGFR was defined as an eGFR < 60 mL/min/1.73 m^2^ [20], and a preserved eGFR was defined as ≥ 60 mL/min/1.73 m^2^. To categorize continuous variables for logistic analysis, we employed the target goals of diabetes care recommended in Taiwan [22]: poor glucose control was defined as fasting glucose ≥ 130 mg/dL (7.2 mmol/L) or HbA1c ≥ 7.0%; hypertension was defined as systolic blood pressure ≥ 130 mmHg, diastolic blood pressure ≥ 80 mmHg, or current use of antihypertensive therapy; hypercholesterolemia was defined as total cholesterol ≥ 160 mg/dL (4.14 mmol/L) or LDL cholesterol ≥ 100 mg/dL (2.59 mmol/L); hypertriglyceridemia was defined as a triglyceride level ≥ 150 mg/dL (1.7 mmol/L); and low HDL cholesterol level was defined as <40 mg/dL (1.0 mmol/L) in men or < 50 mg/dL (1.3 mmol/L) in women. Other continuous variables, including age, diabetes duration, body mass index (BMI), and GPT, were categorized based on the median values.

### 2.4. Statistical Analysis

Continuous variables are expressed as median (interquartile range [IQR]), and categorical data are presented as number (percentage). The Mann–Whitney U test was used to examine the statistical significance of continuous variables between two groups and the Kruskal–Wallis test was used to examine statistical significance of continuous variables among the three groups. The chi-square test was used to examine the statistical significance of categorical variables. Receiver operating characteristic (ROC) curves were generated to differentiate peripheral neuropathic pain based on the eGFR, UACR, and regression model. To identify independent factors associated with peripheral neuropathic pain, univariable logistic regression analysis was used to select potential associated factors (*p* < 0.05), and these potential associated factors were subsequently included in the multivariable logistic regression analyses. The variance inflation factor (VIF) was assessed to examine multicollinearity among the independent variables in the multivariable regression models. For the sensitivity analysis, we included all assessed variables in the multivariable regression analysis. Furthermore, we examined the difference in the prevalence of peripheral neuropathic pain between the reduced and preserved eGFR groups using a UACR-stratified analysis. All statistical tests were two-tailed, and a *p* value < 0.05 was considered statistically significant. Statistical analyses were performed using SPSS software (version 22.0; IBM, Armonk, NY, USA).

## 3. Results

Among the 2365 enrolled patients, 553 (23%) were categorized into the reduced eGFR group and 1812 (77%) into the preserved eGFR group. Table 1 summarizes the clinical characteristics of the patients in the two groups. In addition to the markedly lower median eGFR in the reduced eGFR group than in the preserved eGFR group (*p* < 0.001), the proportions of patients with increased albuminuria were significantly greater in the reduced eGFR group (*p* < 0.001). The proportion of patients with peripheral neuropathic pain, defined as ID pain scores ≥ 2, was significantly greater among those with reduced eGFR than among their preserved eGFR counterparts (28.9% vs.19.8%, *p* < 0.001). The prevalence of peripheral neuropathic pain, defined as DN4 scores ≥ 4, was significantly higher among patients with reduced eGFR than among those with preserved eGFR (12.7% vs. 6.5%, *p* < 0.001).

As the questionnaire scores for peripheral neuropathic pain were significantly different between the reduced and preserved eGFR groups, we examined the relationship between increased albuminuria and peripheral neuropathic pain using eGFR-stratified analyses (Table 2). The distribution of the 1812 patients with preserved eGFR based on the UACR was as follows: normal UACR (*n* = 1261, 69.6%), moderately increased albuminuria (*n* = 418, 23.1%), and severely increased albuminuria (*n* = 133, 7.3%). The prevalence of patients with ID Pain scores ≥ 2 showed a positive trend toward high UACR categories, rising from 15.9% in the normal UACR group to 27.0% in the moderately increased albuminuria group and 33.8% in the severely increased albuminuria group (*p* < 0.001). Similarly, the prevalence of patients with DN4 scores ≥ 4 increased progressively with higher UACR categories, rising from 4.9% in the normal UACR group to 8.9% in the moderately increased albuminuria group and 13.5% in the severely increased albuminuria group (*p* < 0.001).

The distribution of the 553 patients with reduced eGFR based on the UACR was as follows: normal UACR (*n* = 176, 31.8%), moderately increased albuminuria (*n* = 226, 40.9%), and severely increased albuminuria (*n* = 151, 27.3%). The prevalence of ID Pain scores ≥ 2 showed a positive trend toward high UACR categories, rising from 25.6% in the normal UACR group to 26.1% in the moderately increased albuminuria group and 37.1% in the severely increased albuminuria group (*p* = 0.035). Similarly, the prevalence of DN4 scores ≥ 4 increased progressively with the UACR category, rising from 9.7% in the normal UACR group to 10.2% in the moderately increased albuminuria group and 19.9% in the severely increased albuminuria group (*p* = 0.004).

According to the ROC curves, the eGFR significantly discriminated between patients with and without ID Pain scores ≥ 2, with an area under the curve (AUC) of 0.576 (95% confidence interval [CI]: 0.548–0.604) and DN4 scores ≥ 4 based on an AUC of 0.622 (95% CI: 0.580–0.664). However, the eGFR did not significantly differentiate ID Pain scores ≥ 2 in the subgroups of the patients with UACR ≥ 300 mg/g and UACR between 30 and 299 mg/g. Moreover, the eGFR did not significantly differentiate DN4 scores ≥ 4 in the subgroups of patients with UACR between 30 and 299 mg/g (Table 3). UACR significantly differentiated ID Pain scores ≥ 2 based on an AUC of 0.617 (95% CI: 0.590–0.644) and DN4 scores ≥ 4 based on an AUC of 0.649 (95% CI: 0.609–0.690). UACR still significantly differentiated peripheral neuropathic pain in the subgroups of the patients categorized by an eGFR of 60 mL/min/1.73 m^2^ (Table 3).

To identify independently associated factors for ID Pain scores ≥ 2, potential associated factors were selected using univariable logistic regression analysis. In addition to the UACR categories, the following factors were statistically significant: reduced eGFR, age, sex, longer diabetes duration, a history of CAD, BMI, HbA1c, low HDL cholesterol, GPT, and use of antiplatelet agents, antihypertensive therapy, insulin, or metformin (Table 4). Because smoking status is an important associated factor for peripheral neuropathic pain [8], we included the aforementioned potential associated factors and current smoking status in a multivariable regression model. VIF data are shown and indicate no significant multicollinearity between the independent variables. Based on the multivariable logistic regression analysis, increased albuminuria remained independently associated with ID Pain scores ≥ 2 (*p* < 0.001). Compared with the normal UACR group, the risk of peripheral neuropathic pain was significantly increased in the moderately increased albuminuria group (adjusted odds ratio [OR]: 1.462; 95% CI: 1.151–1.857; *p* = 0.002) and in the severely increased albuminuria group (adjusted OR: 2.016; 95% CI: 1.471–2.763; *p* < 0.001). However, a reduced eGFR was not independently associated with peripheral neuropathic pain (*p* = 0.466).

To replicate the above findings, we intended to identify the association between the UACR and peripheral neuropathic pain defined by DN4 scores ≥ 4. In the univariable logistic regression analysis, the potential associated factors for DN4 scores ≥ 4 included increased albuminuria, reduced eGFR, age, sex, diabetes duration, BMI, HbA1c, HDL cholesterol level, GPT, and use of antiplatelet agents, antihypertensive therapy, insulin, or metformin. After adjusting for potential confounders in the multivariable model, increased albuminuria remained independently associated with peripheral neuropathic pain (*p* < 0.001). Compared to the normal UACR group, moderately increased albuminuria did not significantly increase the risk of peripheral neuropathic pain (adjusted OR: 1.352, 95% CI: 0.933–1.958, *p* = 0.111), but severely increased albuminuria was independently associated with peripheral neuropathic pain (adjusted OR: 2.354, 95% CI: 1.524–3.634, *p* < 0.001). However, a reduced eGFR was not independently associated with peripheral neuropathic pain (*p* = 0.294, Table 4).

Table 5 shows the performance of the multivariable logistic regression analysis to differentiate peripheral neuropathic pain based on the ROC curve. There was no significant interaction between UACR and reduced eGFR based on the variable (UACR × reduced eGFR) in the logistic regression analysis (Table 6). For sensitivity analyses, we further examine the association of peripheral neuropathic pain with the continuous data of UACR and eGFR. In addition to the categorial variables in Table 2, continuous data of the variables including age, diabetes duration, BMI, blood pressure, glucose, HbA1c, lipid profile, and GPT were used as confounders in the multivariable sensitivity model. We observed that UACR but not eGFR was independently associated with peripheral neuropathic pain after adjusting for confounding factors (Table 7).

Taken together, these findings revealed that increased albuminuria but not reduced eGFR was independently associated with peripheral neuropathic pain after adjusting for confounding factors. To further understand the relationship between reduced eGFR and peripheral neuropathic pain, we compared the prevalence of peripheral neuropathic pain between the reduced and preserved eGFR groups based on the UACR-stratified analysis. The prevalence of patients with ID Pain scores ≥ 2 was significantly different between the reduced and preserved eGFR groups only within the normal UACR stratification (25.6% vs. 15.9%, *p* = 0.001), but it was not significantly different within the moderately (26.1% vs. 27.0%, *p* = 0.800) or severely (37.1% vs. 33.8%, *p* = 0.568) increased albuminuria stratification (Figure 1). Similarly, the prevalence of patients with DN4 scores ≥ 4 was significantly different between the reduced and preserved eGFR groups only within the normal UACR stratification (9.7% vs. 4.9%, *p* = 0.010) but was not significantly different within the moderately (10.2% vs. 8.9%, *p* = 0.581) or severely (19.9% vs. 13.5%, *p* = 0.155) increased albuminuria stratification (Figure 2).

## 4. Discussion

The main finding of our study was that the severity of albuminuria was significantly associated with peripheral neuropathic pain assessed using questionnaires in patients with T2D. Although a higher prevalence of peripheral neuropathic pain was observed in patients with reduced eGFR than in patients with preserved eGFR, increased albuminuria but not reduced eGFR was independently associated with peripheral neuropathic pain in the multivariable regression analyses. Reduced eGFR was significantly associated with peripheral neuropathic pain only among patients without increased albuminuria. Gao et al. [14] reported that the serum creatinine level was higher in patients with slower NCV and that this level was not an independent factor for abnormal NCV in Chinese patients with T2D. In line with our findings, 24 h urine albumin, but not serum creatinine, was significantly associated with peripheral neuropathic pain in Chinese patients with T2D according to factor selection using least absolute shrinkage and selection operator regression [23]. Because of the close association between albuminuria and reduced eGFR, the high prevalence of peripheral neuropathic pain in patients with reduced eGFR may be a result of increased albuminuria. In the present study, the influence of reduced eGFR on neuropathic pain was significant only in patients without increased albuminuria; however, increased albuminuria was significantly associated with neuropathic pain in patients with reduced and preserved eGFR. Endothelial dysfunction and inflammation, which drive diabetic microvascular disease, can trigger immune activation and neuronal oxidative stress that may be involved in the mechanism underlying albuminuria and peripheral neuropathic pain [24].

The prevalence of peripheral neuropathic pain is underestimated in clinical health care. Although 83% of patients with DM had peripheral neuropathic pain, only 41% had been diagnosed with DPN [12]. Most previous studies were conducted to determine the associated factors for neuropathic pain in patients with established DPN [25]. However, the pathophysiological mechanisms may differ between the sensory and motor manifestations of DPN [9]. We identified the independently associated factors, including increased albuminuria, female sex, BMI, and GPT, for peripheral neuropathic pain based on the ID Pain score and replicated the results using the DN4 score. We previously reported that peripheral neuropathic pain is predictive of an increased risk of total mortality in patients with T2D [16]. Different to the above study, the aim of this retrospective cross-sectional analysis was to reveal the factors associated with peripheral neuropathic pain and promote screening for peripheral neuropathic pain not only among patients with established DPN but also among all patients with T2D, particularly those with the aforementioned independent associated factors. Although the patients were enrolled from the same population [16], we decreased the eGFR threshold of exclusion criterion to 5 mL/min/1.73 m^2^ in patients without renal re-placement therapy to increase the number of patients with a reduced eGFR in this cross-sectional study.

Peripheral neuropathic pain is a syndrome characterized by clinical manifestations of pain associated with lesions or diseases of the somatosensory nervous system. Pain may result from damage to the peripheral somatosensory system attributable to diabetes; however, the exact underlying mechanisms remain unknown [9]. In addition to the known factors associated with DPN [26], dysregulated expression of voltage-gated sodium channels, which play a crucial role in nociceptor function, may be involved in the mechanisms underlying peripheral neuropathic pain [27]. Albuminuria can be induced by inhibiting the expression of the transmembrane protein 63C in renal and human embryonic kidney 293 cells, which widely express voltage-gated sodium channels [28]. Recently, Gylfadottir et al. [29] reported that high macrophage density in the skin was associated with neuropathic pain in patients with T2D and DPN. The mechanism underlying peripheral neuropathic pain might involve macrophage infiltration, which in the kidneys was reported to be associated with an increased urinary albumin excretion rate in diabetic mice [30]. Therefore, inflammation and active immune reactions play important roles in peripheral neuropathic pain and albuminuria [24].

Albuminuria, associated with endothelial dysfunction, is an important factor for vascular complications [31]. In addition to nephropathy and cardiovascular disease, muscle-strength weakening and frailty are associated with albuminuria in older people [32,33]. Alonso-Bouzon et al. [34] reported that frailty was associated with endothelial dysfunction reflected by high blood levels of asymmetric dimethylarginine. Furthermore, albuminuria is associated with cognitive dysfunction in elderly people with frailty, and endothelial dysfunction may play an important role in the mechanisms involved in cognitive decline [35,36]. Cigarette smoking, as a risk factor for endothelial dysfunction, is associated with painful DPN [37,38,39]. However, current smoking was not significantly associated with peripheral neuropathic pain in our univariable logistic regression analysis; it was independently associated with ID Pain scores ≥ 2 but not DN4 scores ≥ 4.

In the present study, males had a lower proportion of peripheral neuropathic pain than females. In line with our findings, Toa et al. [25] reported that female sex was an important associated factor for pain in patients with DPN. Notably, the female sex is a protective factor against DPN, but it was found to be associated with neuropathic pain in patients with DPN [40]. Similarly, in patients with T2D, females reported greater pain intensity than males [41]. Although sex hormones contribute to pain modulation [42], this cannot fully explain the finding that pain dominantly affected female patients in the present study population with a median age of 62 years. Sex differences in neuroimmune and endocrine systems are involved in the modulation of pain signaling [43]. Sieberg et al. [44] reported that female mice had lower plasma corticosterone levels and more anxiety behaviors than male mice in the animal study and that female patients had higher anxiety scores than male patients in response to pain stress in patients with DPN.

In two studies conducted in Asia with patients having T2D and DPN, BMI was not significantly associated with painful DPN [8,11]. Recently, Reynolds et al. [45] reported that BMI was significantly associated with DPN but was not significantly associated with pain in patients with DPN based on the data from the UK Biobank. In the present study, BMI ≥ 25 kg/m^2^ was independently associated with peripheral neuropathic pain. In line with our findings, a meta-analysis showed that obesity (BMI ≥ 30 kg/m^2^) was significantly associated with neuropathic pain in patients with T2D and DPN [25]. A high-fat diet increases neuronal inflammation and leads to an increase in nociceptive nerve fibers in mice [46]. Furthermore, obesity can induce endoplasmic reticulum stress in sensory neurons and allodynia in mice fed a high-fat diet [47]. Low GPT levels were also associated with peripheral neuropathic pain in the present study. Liver steatosis and fibrosis have been reported to be associated with DPN in patients with T2D [48]. However, we did not assess viral hepatitis as a potential confounder in this study.

Insulin and oral insulin secretagogues have been reported to increase the risk of DPN [49]. In the present study, insulin, but not oral antidiabetic drugs, was found to be independently associated with peripheral neuropathic pain. In line with our study, Zhou et al. [50] reported in their meta-analysis that oral antidiabetic drugs were not associated with peripheral neuropathic pain. However, the clinical assessments were performed in 2013, and glucagon-like peptide-1 receptor agonists (GLP-1RAs) and sodium-glucose cotransporter 2 (SGLT2) inhibitors, the new classes of anti-diabetic drugs with cardiorenal benefits, were not assessed in the present study. GLP-1RAs reduce inflammation and oxidative stress by inhibiting astrocyte and microglial activity, improving neuropathic pain, as evidenced by increases in the mechanical paw withdrawal threshold and thermal paw withdrawal latency in a rat model [51]. However, GLP-1RAs did not improve the symptoms of pain or electrophysiological measures in a clinical study of patients with T2D and DPN [52]. SGLT2 inhibitors have been reported to improve neuropathic pain, as represented by an increase in the paw withdrawal threshold [53]. Vafaeinasab et al. [54] reported that SGLT2 inhibitors improved self-report Michigan Neuropathy Screening Instrument scores and the NCV in a double-blind, randomized study of patients with T2D.

The strength of our study lies in determining the associated factors for peripheral neuropathic pain in patients with T2D, not comparing factors between painful and other types of DPN among patients with established DPN. Painful DPN is a syndrome with multiple underlying mechanisms and the painful phenotype is a target for its treatment [9,55]. Because the pathophysiology and treatment of peripheral neuropathic pain are different from those of other types of DPN, screening for peripheral neuropathic pain should not be performed only in patients with established DPN, which has been underdiagnosed in clinical settings [12]. However, this clinical, cross-sectional study has some limitations. First, we did not investigate the underlying mechanisms of increased albuminuria and peripheral neuropathic pain. Second, we could not determine the causal relationship between increased albuminuria and peripheral neuropathic pain. Third, all participants were enrolled from the Pay-for-Performance Program in an outpatient setting. Although the prevalence of diabetic neuropathy was not significantly different in 2013 between the patients in the Pay-for-Performance Program and those not in the program, the prevalence of other complications in these patients might differ from that in patients not enrolled in the program according to the National Health Insurance Research Database in Taiwan [17]. Therefore, the results of this study cannot be generalized to other populations due to a potential selection bias. Further studies for external validity are warranted.

## 5. Conclusions

Among patients with T2D, increased albuminuria was independently associated with peripheral neuropathic pain in both patients with reduced eGFR and those with preserved eGFR. However, reduced eGFR was not independently associated with peripheral neuropathic pain after adjusting for confounding factors, including the UACR category, and the association between reduced eGFR and peripheral neuropathic pain was statistically significant only in patients with a normal UACR. However, the underlying mechanisms and causal effects of increased albuminuria and peripheral neuropathic pain require further investigation.

## Figures and Tables

**Figure 1:**
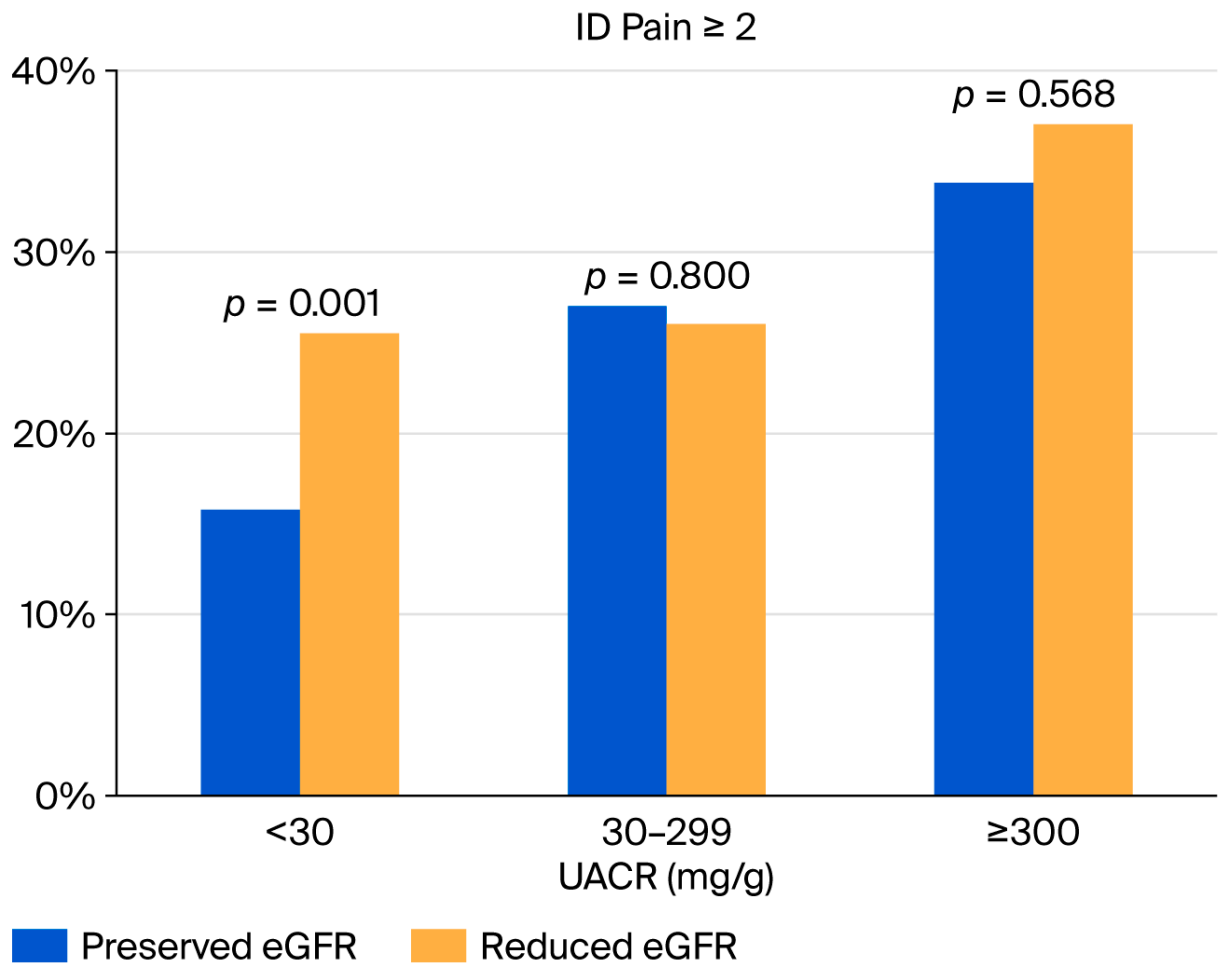
Prevalence of patients with ID Pain score ≥ 2 based on a UACR-stratified analysis. The prevalence of peripheral neuropathic pain was significantly different between the preserved and reduced eGFR groups only within the normal UACR (<30 mg/g) stratification but was not significantly different within the moderately (30–299 mg/g) or severely increased albuminuria (≥300 mg/g) stratification. Abbreviations: ID Pain, Identification Pain; UACR, urinary albumin-to-creatinine ratio.

**Figure 2:**
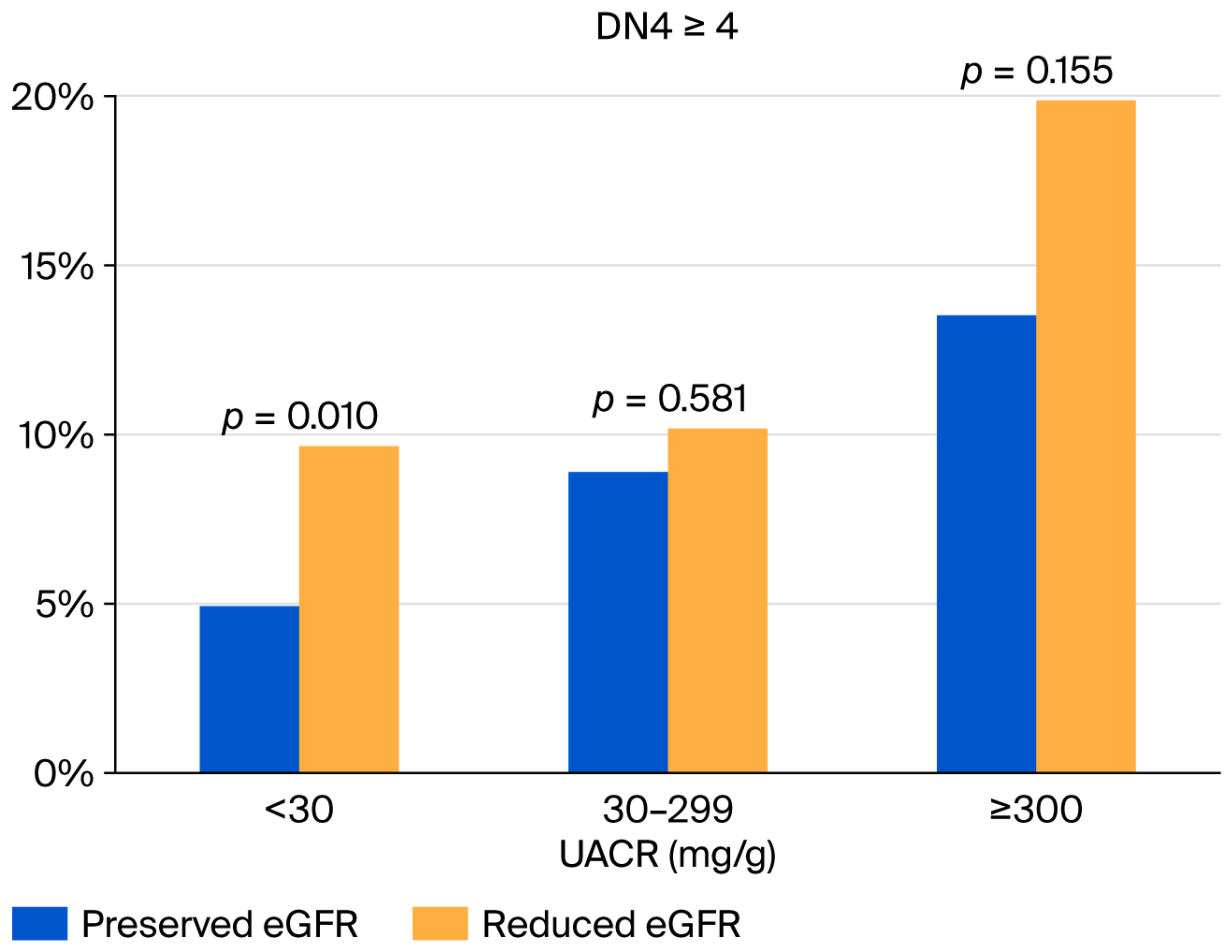
Prevalence of patients with DN4 scores ≥ 4 based on a UACR-stratified analysis. The prevalence of peripheral neuropathic pain was significantly different between the preserved and reduced eGFR groups only within the normal UACR (<30 mg/g) stratification but was not significantly different within the moderately (30–299 mg/g) or severely increased albuminuria (≥300 mg/g) stratification. Abbreviations: DN4, Douleur Neuropathique en 4; UACR, urinary albumin-to-creatinine ratio.

**Table 1:** Characteristics of patients categorized by an eGFR of 60 mL/min/1.73 m^2^.

	Reduced eGFR (*n* = 553)		Preserved eGFR (*n* = 1812)		*p*
	Median	(IQR)	Median	(IQR)	
Demographic information					
Age (year)	73	(65, 81)	60	(53, 69)	<0.001
Male, *n* (%)	330	(59.7%)	1001	(55.2%)	0.066
Diabetes duration (year)	12.6	(6.9, 20.7)	7.4	(3.2, 12.7)	<0.001
Current smoker, *n* (%)	32	(5.8%)	207	(11.4%)	<0.001
Hypertension, *n* (%)	537	(97.1%)	1519	(83.8%)	<0.001
CAD history, *n* (%)	242	(43.8%)	471	(26.0%)	<0.001
BMI (kg/m^2^)	25.0	(22.8, 27.5)	25.4	(23.1, 28.0)	0.011
Systolic BP (mmHg)	130	(126, 140)	130	(125, 136)	<0.001
Diastolic BP (mmHg)	78	(70, 80)	78	(74, 80)	0.001
Biochemical data					
Fasting glucose (mmol/L)	7.4	(6.1, 9.2)	7.8	(6.6, 9.8)	<0.001
HbA1c (%)	7.2	(6.5, 8.2)	7.4	(6.7, 8.6)	<0.001
Total cholesterol (mmol/L)	4.2	(3.7, 4.8)	4.3	(3.8, 4.9)	0.009
LDL cholesterol (mmol/L)	2.5	(2.1, 3.1)	2.7	(2.1, 3.2)	0.009
HDL cholesterol (mmol/L)	1.2	(1.0, 1.5)	1.3	(1.1, 1.5)	<0.001
Triglycerides (mmol/L)	1.4	(0.9, 2.1)	1.3	(0.9, 1.9)	0.263
GPT (U/L)	21	(16, 29)	26	(19, 40)	<0.001
eGFR (mL/min/1.73 m^2^)	42.7	(31.6, 53.4)	94.0	(79.4, 104.0)	<0.001
UACR (mg/g)	85.3	(18.8, 381.7)	10.1	(3.6, 42.5)	<0.001
UACR (category)					<0.001
<30 mg/g	176	(31.8%)	1261	(69.6%)	
30–299 mg/g	226	(40.9%)	418	(23.1%)	
≥300 mg/g	151	(27.3%)	133	(7.3%)	
Peripheral neuropathic pain					
ID Pain score ≥ 2, *n* (%)	160	(28.9%)	358	(19.8%)	<0.001
DN4 score ≥ 4, *n* (%)	70	(12.7%)	117	(6.5%)	<0.001
Medications					
Antiplatelet drugs, *n* (%)	226	(40.9%)	448	(24.7%)	<0.001
Statins, *n* (%)	373	(67.5%)	1154	(63.7%)	0.105
Fenofibrates, *n* (%)	65	(11.8%)	149	(8.2%)	0.011
Antihypertensive drugs, *n* (%)	471	(85.2%)	1051	(58.0%)	<0.001
Insulin therapy, *n* (%)	199	(36.0%)	429	(23.7%)	<0.001
Oral antidiabetic drugs, *n* (%)	467	(84.4%)	1681	(92.8%)	<0.001
Insulin secretagogues	282	(51.0%)	924	(51.0%)	0.999
Metformin	171	(30.9%)	1082	(59.7%)	<0.001
Thiazolidinediones	121	(21.9%)	375	(20.7%)	0.589
Alpha-glucosidase inhibitors	105	(19.0%)	230	(12.7%)	<0.001
DPP4 inhibitors	264	(47.7%)	823	(45.4%)	0.363

Abbreviations: BMI, body mass index; BP, blood pressure; CAD, coronary artery disease; DN4, Douleur Neuropathique en 4; DPP4, dipeptidyl peptidase-4; eGFR, estimated glomerular filtration rate; GPT, glutamate pyruvate transaminase; HbA1c, glycated hemoglobin; HDL, high-density lipoprotein; ID Pain, Identification Pain; IQR, interquartile range; LDL, low-density lipoprotein; UACR, urinary albumin-to-creatinine ratio.

**Table 2:** Characteristics of patients categorized by the UACR in the preserved and reduced eGFR groups.

Preserved eGFR (eGFR ≥ 60 mL/min/1.73 m^2^)							
	UACR						*p*
	<30 mg/g (*n* = 1261)		30–299 mg/g (*n* = 418)		≥300 mg/g (*n* = 133)		
	Median	(IQR)	Median	(IQR)	Median	(IQR)	
Demographic information							
Age (year)	60	(52, 67)	62	(54, 73)	59	(53, 70)	<0.001
Male, *n* (%)	702	(55.7%)	224	(53.6%)	75	(56.4%)	0.768
Diabetes duration (year)	6.6	(2.8, 12.1)	8.7	(3.8, 14.7)	9.4	(5.0, 14.9)	<0.001
Current smoker, *n* (%)	132	(10.5%)	56	(13.4%)	19	(14.3%)	0.058
Hypertension, *n* (%)	1019	(80.8%)	370	(88.5%)	130	(97.7%)	<0.001
CAD history, *n* (%)	313	(24.8%)	122	(29.2%)	36	(27.1%)	0.160
BMI (kg/m^2^)	25.2	(23.0, 27.7)	25.7	(23.1, 28.5)	26.4	(23.7, 29.4)	0.002
Systolic BP (mmHg)	130	(124, 135)	130	(126, 138)	132	(126, 142)	<0.001
Diastolic BP (mmHg)	78	(73, 80)	78	(75, 80)	80	(77, 85)	0.004
Peripheral neuropathic pain							
ID Pain score ≥ 2, *n* (%)	200	(15.9%)	113	(27.0%)	45	(33.8%)	<0.001
DN4 score ≥ 4, *n* (%)	62	(4.9%)	37	(8.9%)	18	(13.5%)	<0.001
Biochemical data							
Fasting glucose (mmol/L)	7.7	(6.6, 9.4)	8.1	(6.7, 10.4)	8.6	(6.5, 11.6)	0.001
HbA1c (%)	7.3	(6.6, 8.3)	7.8	(6.8, 9.0)	8.1	(7.0, 9.5)	<0.001
Total cholesterol (mmol/L)	4.3	(3.8, 5.0)	4.3	(3.7, 4.9)	4.5	(3.9, 5.3)	0.034
LDL cholesterol (mmol/L)	2.7	(2.1, 3.2)	2.6	(2.1, 3.1)	2.9	(2.3, 3.4)	0.008
HDL cholesterol (mmol/L)	1.3	(1.1, 1.6)	1.2	(1.0, 1.5)	1.2	(1.0, 1.4)	<0.001
Triglycerides (mmol/L)	1.3	(0.9, 1.9)	1.4	(1.0, 1.9)	1.4	(1.0, 1.9)	0.872
GPT (U/L)	26	(19, 40)	26	(18, 39)	29.0	(21, 43)	0.193
eGFR (mL/min/1.73 m^2^)	95.8	(82.2, 104.6)	90.0	(75.5, 101.9)	88.1	(73.0, 101.1)	<0.001
UACR (mg/g)	5.1	(2.8, 11.3)	81.7	(43.5, 164.0)	706.0	(456.3, 1309.5)	<0.001
Medications							
Antiplatelet drugs, *n* (%)	278	(22.0%)	125	(29.9%)	45	(33.8%)	<0.001
Statins, *n* (%)	784	(62.2%)	266	(63.6%)	104	(78.2%)	0.002
Fenofibrate, *n* (%)	92	(7.3%)	34	(8.1%)	23	(17.3%)	0.001
Antihypertensive drugs, *n* (%)	646	(51.2%)	291	(69.6%)	114	(85.7%)	<0.001
Insulin therapy, *n* (%)	254	(20.1%)	121	(28.9%)	54	(40.6%)	<0.001
Oral antidiabetic drugs, *n* (%)	1175	(93.2%)	385	(92.1%)	121	(91.0%)	0.268
Insulin secretagogues	620	(49.2%)	232	(55.5%)	72	(54.1%)	0.039
Metformin	774	(61.4%)	234	(56.0%)	74	(55.6%)	0.040
Thiazolidinediones	263	(20.9%)	81	(19.4%)	31	(23.3%)	0.892
Alpha-glucosidase inhibitors	133	(10.5%)	77	(18.4%)	20	(15.0%)	<0.001
DPP4 inhibitors	528	(41.9%)	231	(55.3%)	64	(48.1%)	<0.001
**Reduced eGFR (eGFR < 60 mL/min/1.73 m^2^)**							
	**UACR**						***p***
	**<30 mg/g (** ***n*** **= 176)**		**30–299 mg/g (** ***n*** **= 226)**		**≥300 mg/g (** ***n*** **= 151)**		
	**Median**	**(IQR)**	**Median**	**(IQR)**	**Median**	**(IQR)**	
Demographic information							
Age (year)	75	(66, 82)	75	(68, 83)	70.0	(61, 77)	<0.001
Male, *n* (%)	105	(59.7%)	135	(59.7%)	90	(59.6%)	1.000
Diabetes duration (year)	12.2	(5.5, 17.4)	12.8	(7.5, 22.4)	14.3	(7.1, 22.4)	0.044
Currently smoking, *n* (%)	9	(5.1%)	13	(5.8%)	10	(6.6%)	0.844
Hypertension, *n* (%)	164	(93.2%)	222	(98.2%)	151	(100.0%)	<0.001
CAD history, *n* (%)	75	(42.6%)	105	(46.5%)	62	(41.1%)	0.546
BMI (kg/m^2^)	24.6	(22.6, 26.9)	24.8	(22.8, 27.6)	25.3	(23.3, 28.2)	0.086
Systolic BP (mmHg)	130	(123, 136)	130	(124, 140)	132	(128, 144)	<0.001
Diastolic BP (mmHg)	78	(70, 80)	78	(70, 80)	79	(74, 84)	0.004
Peripheral neuropathic pain							
ID Pain score ≥ 2, *n* (%)	45	(25.6%)	59	(26.1%)	56	(37.1%)	0.035
DN4 score ≥ 4, *n* (%)	17	(9.7%)	23	(10.2%)	30	(19.9%)	0.007
Biochemical data							
Fasting glucose (mmol/L)	7.5	(6.4, 9.2)	7.2	(5.8, 9.1)	7.6	(6.0, 9.4)	0.302
HbA1c (%)	7.3	(6.7, 8.4)	7.0	(6.3, 8.2)	7.2	(6.6, 8.1)	0.046
Total cholesterol (mmol/L)	4.1	(3.7, 4.8)	4.4	(3.7, 4.9)	4.1	(3.6, 4.7)	0.252
LDL cholesterol (mmol/L)	2.5	(2.1, 3.1)	2.7	(2.2, 3.1)	2.4	(1.9, 3.1)	0.111
HDL cholesterol (mmol/L)	1.2	(1.0, 1.5)	1.2	(1.0, 1.5)	1.1	(0.9, 1.4)	0.061
Triglycerides (mmol/L)	1.3	(0.9, 2.1)	1.4	(1.0, 2.1)	1.3	(0.9, 2.1)	0.718
GPT (U/L)	22	(16, 30)	21	(16, 29)	20	(16, 29)	0.319
eGFR (mL/min/1.73 m^2^)	49.7	(39.2, 55.9)	41.7	(31.2, 53.2)	36.2	(24.3, 48.0)	<0.001
UACR (mg/g)	10.2	(4.0, 18.0)	97.0	(53.4, 199.1)	1131.0	(570.6, 2231.0)	<0.001
Medications							
Antiplatelet drugs, *n* (%)	65	(36.9%)	93	(41.2%)	68	(45.0%)	0.330
Statins, *n* (%)	120	(68.2%)	143	(63.3%)	110	(72.8%)	0.146
Fenofibrate, *n* (%)	18	(10.2%)	28	(12.4%)	19	(12.6%)	0.747
Antihypertensive drugs, *n* (%)	139	(79.0%)	195	(86.3%)	137	(90.7%)	0.010
Insulin therapy, *n* (%)	45	(25.6%)	79	(35.0%)	75	(49.7%)	<0.001
Oral antidiabetic drugs, *n* (%)	163	(92.6%)	180	(79.6%)	124	(82.1%)	0.001
Insulin secretagogues	100	(56.8%)	109	(48.2%)	73	(48.3%)	0.173
Metformin	71	(40.3%)	67	(29.6%)	33	(21.9%)	0.001
Thiazolidinediones	45	(25.6%)	47	(20.8%)	29	(19.2%)	0.335
Alpha-glucosidase inhibitors	31	(17.6%)	43	(19.0%)	31	(20.5%)	0.799
DPP4 inhibitors	87	(49.4%)	101	(44.7%)	76	(50.3%)	0.484

Abbreviations: BMI, body mass index; BP, blood pressure; CAD, coronary artery disease; DN4, Douleur Neuropathique en 4; DPP4, dipeptidyl peptidase-4; eGFR, estimated glomerular filtration rate; GPT, glutamate pyruvate transaminase; HbA1c, glycated hemoglobin; HDL, high-density lipoprotein; ID Pain, Identification Pain; IQR, interquartile range; LDL, low-density lipoprotein; UACR, urinary albumin-to-creatinine ratio.

**Table 3:** Receiver operating characteristic (ROC) curves to differentiate peripheral neuropathic pain based on eGFR or UACR.

eGFR for Differentiating Peripheral Neuropathic Pain			
	ROC Curve		
	AUC	(95% CI)	*p*
ID Pain score ≥ 2			
In all patients	0.576	(0.548, 0.604)	<0.001
In the UACR subgroups			
<30 mg/g	0.553	(0.512, 0.594)	0.008
30–299 mg/g	0.515	(0.466, 0.565)	0.558
≥300 mg/g	0.538	(0.469, 0.607)	0.289
DN4 score ≥ 4			
In all patients	0.622	(0.580, 0.664)	<0.001
In the UACR subgroups			
<30 mg/g	0.590	(0.525, 0.656)	0.007
30–299 mg/g	0.558	(0.483, 0.632)	0.142
≥300 mg/g	0.591	(0.504, 0.679)	0.046
**UACR for Differentiating Peripheral Neuropathic Pain**			
	**ROC Curve**		
	**AUC**	**(95% CI)**	***p***
ID Pain score ≥ 2			
In all patients	0.617	(0.590, 0.644)	<0.001
In the eGFR subgroups			
≥60 mL/min/1.73 m^2^	0.616	(0.584, 0.649)	<0.001
<60 mL/min/1.73 m^2^	0.556	(0.503, 0.609)	0.038
DN4 score ≥ 4			
In all patients	0.649	(0.609, 0.690)	<0.001
In the eGFR subgroups			
≥60 mL/min/1.73 m^2^	0.635	(0.585, 0.685)	<0.001
<60 mL/min/1.73 m^2^	0.602	(0.530, 0.675)	0.006

Abbreviations: AUC, area under the curve; CI, confidence interval; DN4, Douleur Neuropathique en 4 questionnaire; eGFR, estimated glomerular filtration rate; ID Pain, Identification Pain questionnaire; UACR, urinary albumin-to-creatinine ratio.

**Table 4:** Logistic regression for the factors associated with peripheral neuropathic pain.

ID Pain Score ≥ 2								
	Univariable				Multivariable			
	*n*	OR	(95% CI)	*p*	Adjusted OR	(95% CI)	*p*	VIF
UACR				<0.001			<0.001	1.243
<30 mg/g	1437	1.000			1.000			
30–299 mg/g	644	1.773	(1.420, 2.214)	<0.001	1.462	(1.151, 1.857)	0.002	
≥300 mg/g	284	2.685	(2.031, 3.550)	<0.001	2.016	(1.471, 2.763)	<0.001	
Reduced eGFR	553	1.654	(1.331, 2.055)	<0.001	1.104	(0.847, 1.438)	0.466	1.374
Age ≥ 63 years *	1187	1.374	(1.130, 1.672)	0.001	1.045	(0.823, 1.327)	0.719	1.387
Male	1331	0.558	(0.458, 0.679)	<0.001	0.516	(0.414, 0.643)	<0.001	1.148
Diabetes duration ≥ 8.43 years *	1183	1.843	(1.510, 2.249)	<0.001	1.422	(1.141, 1.773)	0.002	1.181
Currently smoking	239	1.018	(0.738, 1.405)	0.914	1.569	(1.099, 2.239)	0.013	1.114
Hypertension	2056	1.247	(0.921, 1.690)	0.154				
CAD history	713	1.403	(1.142, 1.724)	0.001	1.126	(0.886, 1.431)	0.334	1.279
BMI ≥ 25 kg/m^2^ *	1184	1.277	(1.050, 1.553)	0.014	1.324	(1.073, 1.633)	0.009	1.076
Systolic BP ≥ 130 mmHg	1335	1.136	(0.932, 1.384)	0.207				
Diastolic BP ≥ 80 mmHg	999	0.924	(0.758, 1.126)	0.432				
Fasting glucose ≥ 7.2 mmol/L	1407	1.062	(0.870, 1.296)	0.555				
HbA1c ≥ 7%	1527	1.269	(1.030, 1.563)	0.025	1.113	(0.888, 1.396)	0.353	1.107
Total cholesterol ≥ 4.1 mmol/L	1388	1.031	(0.846, 1.257)	0.763				
LDL cholesterol ≥ 2.59 mmol/L	1246	1.011	(0.832, 1.229)	0.913				
Low HDL cholesterol level	788	1.318	(1.077, 1.614)	0.007	1.012	(0.815, 1.258)	0.912	1.079
Triglycerides ≥ 1.7 mmol/L	820	0.950	(0.773, 1.167)	0.625				
GPT ≥ 25 U/L *	1209	0.701	(0.576, 0.853)	<0.001	0.785	(0.635, 0.971)	0.026	1.109
Antiplatelet drugs	674	1.492	(1.212, 1.836)	<0.001	1.243	(0.975, 1.585)	0.080	1.288
Statins	1527	1.039	(0.847, 1.275)	0.713				
Fenofibrates	214	1.194	(0.861, 1.655)	0.289				
Antihypertensive drugs	1522	1.600	(1.292, 1.983)	<0.001	1.085	(0.847, 1.392)	0.518	1.284
Insulin therapy	628	1.906	(1.548, 2.347)	<0.001	1.492	(1.182, 1.882)	<0.001	1.167
Oral antidiabetic drugs	2148	0.986	(0.704, 1.381)	0.935				
Insulin secretagogues	1206	0.979	(0.806, 1.190)	0.831				
Metformin	1253	0.754	(0.621, 0.917)	0.005	0.933	(0.756, 1.152)	0.520	1.092
Thiazolidinediones	496	1.051	(0.829, 1.333)	0.681				
Alpha-glucosidase inhibitors	335	1.301	(0.997, 1.699)	0.053				
DPP4 inhibitors	1087	1.082	(0.890, 1.315)	0.430				
**DN4 Score ≥ 4**								
	**Univariable**				**Multivariable**			
	***n***	**OR**	**(95% CI)**	***p***	**Adjusted OR**	**(95% CI)**	***p***	**VIF**
UACR				<0.001			<0.001	1.241
<30 mg/g	1437	1.000			1.000			
30–299 mg/g	644	1.766	(1.245, 2.505)	0.001	1.352	(0.933, 1.958)	0.111	
≥300 mg/g	284	3.496	(2.380, 5.135)	<0.001	2.354	(1.524, 3.634)	<0.001	
Reduced eGFR	553	2.100	(1.535, 2.871)	<0.001	1.225	(0.839, 1.788)	0.294	1.372
Age ≥ 63 years *	1187	1.819	(1.335, 2.479)	<0.001	1.422	(0.984, 2.056)	0.061	1.371
Male	1331	0.638	(0.473, 0.861)	0.003	0.689	(0.495, 0.958)	0.027	1.147
Diabetes duration ≥ 8.43 years *	1183	1.976	(1.446, 2.701)	<0.001	1.361	(0.968, 1.914)	0.077	1.181
Currently smoking	239	0.820	(0.482, 1.395)	0.464	1.237	(0.695, 2.200)	0.470	1.113
Hypertension	2056	1.661	(0.981, 2.814)	0.059				
CAD history	713	1.358	(0.994, 1.855)	0.054				
BMI ≥ 25 kg/m^2^ *	1184	1.367	(1.011, 1.849)	0.042	1.430	(1.040, 1.967)	0.028	1.076
Systolic BP ≥ 130 mmHg	1335	1.111	(0.821, 1.505)	0.495				
Diastolic BP ≥ 80 mmHg	999	1.000	(0.739, 1.353)	0.999				
Fasting glucose ≥ 7.2 mmol/L	1407	1.304	(0.954, 1.783)	0.096				
HbA1c ≥ 7%	1527	1.699	(1.207, 2.391)	0.002	1.638	(1.140, 2.354)	0.008	1.107
Total cholesterol ≥ 4.1 mmol/L	1388	1.006	(0.743, 1.363)	0.969				
LDL cholesterol ≥ 2.59 mmol/L	1246	0.988	(0.733, 1.332)	0.937				
Low HDL cholesterol level	788	1.708	(1.263, 2.309)	0.001	1.329	(0.967, 1.828)	0.080	1.079
Triglycerides ≥ 1.7 mmol/L	820	0.953	(0.695, 1.307)	0.765				
GPT ≥ 25 U/L *	1209	0.616	(0.455, 0.836)	0.002	0.703	(0.508, 0.975)	0.034	1.109
Antiplatelet drugs	674	1.520	(1.113, 2.076)	0.008	1.158	(0.825, 1.627)	0.396	1.139
Statins	1527	0.957	(0.701, 1.306)	0.782				
Fenofibrates	214	1.459	(0.920, 2.313)	0.108				
Antihypertensive drugs	1522	1.825	(1.290, 2.580)	0.001	1.076	(0.726, 1.595)	0.715	1.259
Insulin therapy	628	1.826	(1.340, 2.490)	<0.001	1.254	(0.890, 1.767)	0.196	1.167
Oral antidiabetic drugs	2148	0.884	(0.538, 1.453)	0.627				
Insulin secretagogues	1206	1.140	(0.845, 1.539)	0.390				
Metformin	1253	0.688	(0.509, 0.929)	0.015	0.904	(0.656, 1.246)	0.538	1.090
Thiazolidinediones	496	1.253	(0.884, 1.775)	0.205				
Alpha-glucosidase inhibitors	335	1.173	(0.780, 1.766)	0.443				
DPP4 inhibitors	1087	1.264	(0.937, 1.704)	0.125				

* Categorized according to the median. Abbreviations: BMI, body mass index; BP, blood pressure; CAD, coronary artery disease; CI, confidence interval; DN4, Douleur Neuropathique en 4 questionnaire; DPP4, dipeptidyl peptidase-4; eGFR, estimated glomerular filtration rate; GPT, glutamate pyruvate transaminase; HbA1c, glycated hemoglobin; HDL, high-density lipoprotein; ID Pain, Identification Pain questionnaire; LDL, low-density lipoprotein; OR, odds ratio; UACR, urinary albumin-to-creatinine ratio; VIF, variance inflation factor.

**Table 5:** The performance of the regression analysis to differentiate peripheral neuropathic pain.

	AUC	(95% CI)	*p*	Sensitivity	Specificity
ID Pain score ≥ 2	0.673	(0.647, 0.699)	<0.001	57.9%	68.1%
DN4 score ≥ 4	0.705	(0.666, 0.743)	<0.001	62.0%	70.7%

Abbreviations: AUC, area under the curve; CI, confidence interval; DN4, Douleur Neuropathique en 4; ID Pain, Identification Pain.

**Table 6:** The interaction between UACR and reduced eGFR status on peripheral neuropathic pain.

	ID Pain Score ≥ 2 *			DN4 Score ≥ 4 ^#^		
	OR	(95% CI)	*p*	OR	(95% CI)	*p*
UACR	1.556	(1.296, 1.868)	<0.001	1.529	(1.161, 2.013)	0.002
Reduced eGFR	1.368	(0.945, 1.982)	0.097	1.259	(0.722, 2.196)	0.417
UACR * reduced eGFR	0.779	(0.573, 1.059)	0.111	0.975	(0.633, 1.502)	0.909

* After adjusting for age, sex, diabetes duration, smoking, CAD history, BMI, HbA1c, HDL cholesterol, GPT, antiplatelet drugs, antihypertensive drugs, insulin therapy, and metformin. ^#^ After adjusting for age, sex, diabetes duration, smoking, BMI, HbA1c, HDL cholesterol, GPT, antiplatelet drugs, antihypertensive drugs, insulin therapy, and metformin. Abbreviations: BMI, body mass index; CAD, coronary artery disease; CI, confidence interval; DN4, Douleur Neuropathique en 4; eGFR, estimated glomerular filtration rate; GPT, glutamate pyruvate transaminase; HbA1c, glycated hemoglobin; HDL, high-density lipoprotein; ID Pain, Identification Pain; OR, odds ratio; UACR, urinary albumin-to-creatinine ratio.

**Table 7:** The regression analysis for sensitivity analysis.

	ID Pain Score ≥ 2			DN4 Score ≥ 4			
	Adjusted OR	(95% CI)	*p*	Adjusted OR	(95% CI)	*p*	VIF
UACR (1000 mg/g) *	1.262	(1.066, 1.494)	0.007	1.441	(1.181, 1.757)	<0.001	1.187
eGFR (mL/min/1.73 m^2^)	0.997	(0.992, 1.002)	0.315	0.996	(0.989, 1.003)	0.287	1.884

After adjusting all assessed variables, including continuous variables (age, diabetes duration, body mass index, systolic and diastolic blood pressure, glucose, glycated hemoglobin, total, low-density lipoprotein, and high-density lipoprotein cholesterol levels, triglycerides, and glutamate pyruvate transaminase) and categorical variables (sex, currently smoking, hypertension, coronary artery disease, use of antiplatelet drugs, statins, fenofibrates, antihypertensive drugs, insulin therapy, any oral antidiabetic drug, insulin secretagogues, metformin, thiazolidinediones, alpha-glucosidase inhibitors, and dipeptidyl peptidase-4 inhibitors). * To facilitate interpretation, the UACR is rescaled to 1000 mg/g. Abbreviations: CI, confidence interval; eGFR, estimated glomerular filtration rate; DN4, Douleur Neuropathique en 4; ID Pain, Identification Pain; OR, odds ratio; UACR, urinary albumin-to-creatinine ratio; VIF, variance inflation factor.

## Data Availability

The data presented in this study are available on request from the corresponding author due to ethical restrictions.

## Abbreviations

The following abbreviations are used in this manuscript:

AUC	area under the curve
BMI	body mass index
CI	confidence interval
CKD-EPI	Chronic Kidney Disease Epidemiology Collaboration
DM	diabetes mellitus
DN4	Douleur Neuropathique en 4
DPN	diabetic peripheral neuropathy
DPP4	dipeptidyl peptidase-4
eGFR	estimated glomerular filtration rate
GLP-1RAs	glucagon-like peptide-1 receptor agonists
GPT	glutamate pyruvate transaminase
HbA1c	glycated hemoglobin
HDL	high-density lipoprotein
ID Pain	Identification Pain
IDF	International Diabetes Federation
IQR	interquartile range
LDL	low-density lipoprotein
NCV	nerve conduction velocity
OR	odds ratio
ROC	receiver operating characteristic
SGLT2	sodium-glucose cotransporter 2
T2D	type 2 diabetes
UACR	urinary albumin-to-creatinine ratio
